# Perioral Dermatitis after Dental Filling in a 12-Year-Old Girl: Involvement of Cholinergic System in Skin Neuroinflammation?

**DOI:** 10.1100/tsw.2008.31

**Published:** 2008-02-06

**Authors:** Fabrizio Guarneri, Herbert Marini

**Affiliations:** ^1^ Department of Territorial Social Medicine, Section of Dermatology, A.O.U. Policlinico “G. Martino”, Via Consolare Valeria-Gazzi, Pad. H, 4th floor, University of Messina, 98125 Messina, Italy; ^2^ Department of Biochemical, Physiological and Nutritional Sciences, Section of Physiology and Human Nutrition, A.O.U. Policlinico “G. Martino”, Via Consolare Valeria-Gazzi, Torre Biologica, 5th floor, University of Messina, 98125 Messina, Italy

**Keywords:** perioral dermatitis, cholinergic system, neurogenic inflammation, mercury, thiomersal

## Abstract

The etiopathogenesis of perioral dermatitis (PD) is still unknown and, consequently, medical treatment is difficult, not precisely defined, and often unsatisfactory. On the basis of a peculiar case that appeared soon after multiple dental fillings with a mercury-containing amalgam, we proposed that neurogenic inflammation could play a role in the pathogenesis of PD. According to the new findings provided by clinical and basic research, neurogenic inflammation has a relevant part in the pathogenesis of many cutaneous diseases. We report a similar case of PD, taking into account, more specifically, the possible involvement of the cholinergic system. Also in this case, PD seems to be mainly related to the mercury contained in dental fillings and/or its organic compounds formed by oral/gut bacteria. We examined the possible role of these substances as causes of PD, providing new information on the possible cross-talk between neuroimmunodermatology and potential triggers of PD.

## INTRODUCTION

Perioral dermatitis (PD) is classically characterized by perioral papules and pustules sparing a narrow zone around the lips. However, clinically polymorphic presentations, including papulovesicles and erythematous-eczcematous and scaling eruption, are frequently reported. Many exogenous (irritants, topical steroids, UV radiations, *Demodex folliculorum*, *Candida albican*s, and other microbes) and endogenous (hormones) factors have been proposed as possible causative agents[1,2,3,4,5,6,7], but the etiopathogenesis is still unknown. Consequently, treatment is difficult and often unsatisfactory.

## CASE REPORT

A Caucasian girl, 12 years old, came to our observation because of erythematous-eczematous, nonitching, perioral lesions, which appeared 6 weeks earlier. Clinical history revealed that the filling of three molars (first upper right and left, second lower right), performed 2 months earlier, had caused mild pain and local inflammation in the filling sites. Complete examination of the oral cavity showed redness and edema surrounding the aforementioned teeth, and a localized pain in the filling sites was still reported by the patient. Composition of the amalgam was 50% mercury, 34.65% silver, 8.95% tin, 5.9% copper, and 0.5% zinc. Use of topical or systemic drugs in the last 3 months, as well as recent (since about 2 weeks before the onset of perioral lesions) prolonged UV exposure, use of cosmetics, or use of toothpaste were denied (the patient reported that oral hygiene was maintained by regular use of toothbrush with water only). Cutaneous manifestations were not related to ingestion of specific foods and no variation in dietary habits in the last months (before or after the onset of PD) was reported. Routine blood exams, thyroid and sex hormones were normal. Lesional microbial and fungal flora were quali-quantitatively normal. Specialist examinations excluded psychological and endocrinological causes. We performed a patch test, using the standard hapten series plus screening series of cosmetics and odontoiatric materials (Table 1, panel A). Patch test reading was performed at days 3, 5, 7, 9, and 10 after application; a single sensitization (++) to thiomersal (sodium ethylmercury thiosalicylate) was revealed at day 3 (72 h) and confirmed at day 5 (120 h). Oral and/or cutaneous biopsy was refused by the patient. In light of available data, we diagnosed PD. We observed that lesions overlapped with the facial areas innervated by infraorbitary nerves and right mental nerve; interestingly, these nerves also innervate the dental half arcs that contained the amalgam-filled molars. Noteworthy, at patch test reading, we noticed new lesions in the facial area innervated by the left mental nerve (Figs. 1a and 2). As expected because of recent onset, the new lesions had a markedly more inflammatory appearance than the chronicized ones. Based on our previous experience[8], we suggested dental filling removal, although amalgam did not contain thiomersal, and the replacement (with acrylates) led to spontaneous healing in 4 weeks (Fig. 1b), without relapses over a 6-month follow-up period. Differential diagnosis in this case could include irritant/allergic contact dermatitis and atopic eczema with exclusively perioral localization. Irritant contact dermatitis was excluded because the quick onset of the lesions was not justified by the use of topical irritants. Allergic contact dermatitis was unlikely, because the patient denied recent use of topical products and, moreover, the patch test (comprising all components of topical products previously used by the patient) was negative. Another possibility considered was the “indirect” contact with materials released by dental amalgam, diluted in saliva, and carried to perioral area by the tongue, by fingers (when touching lips or tongue and, successively, facial skin), while brushing teeth, or during sleep. In all the above cases, however, extensive involvement of the oral mucosa and/or cheilitis, as well as irregular (asymmetric) distribution of perioral lesions, would be expected. However, to exclude this possibility definitively, we successively patch tested the patient again, using all the materials contained in the amalgam (including those less frequently responsible for allergy) and related compounds (Table 1, panel B), and the new test gave negative results.

Clinical features of lesions, family history (atopic father), and sensitization to thiomersal could support the diagnosis of atopic eczema with exclusively perioral localization, but the patient had neither the typical facial features of atopic subjects nor a history of allergic diseases, laboratory parameters were negative, and the lesions were nonitching. The quick and complete resolution after amalgam removal was, however, decisive to exclude atopic eczema.

## DISCUSSION

The nervous system takes active part in the pathomechanism of several skin diseases (psoriasis, atopic dermatitis, urticaria, contact hypersensitivity)[9]. Interplay of neurons, keratinocytes, and Langerhans’ cells, releasing acetylcholine and expressing acetylcholine receptors, is critical in mucocutaneous homeostasis and

**TABLE 1**
**Haptens Used for First (A) and Second (B) Patch Test in our Patient.**

| **(A) First Patch Test** | |
|---|---|
| Thiuram mix 1% | Neomycin sulfate 20% |
| Nickel sulfate 5% | Colophony 20% |
| Phenol-formaldehyde resin 5% | Ethylenediamine 1% |
| Balsam of Peru 25% | Fragrance mix 8% |
| Lanolin alcohols 30% | Potassium dichromate 0.5% |
| p-Phenylenediamine 1% | Mercaptobenzothiazole 2% |
| Epoxy resin 1% | Disperse blue 1% |
| Disperse yellow 1% | Disperse red 1% |
| Disperse orange 1% | Paraben mix 15% |
| Kathon CG 0.01% (in water) | Formaldehyde 1% (in water) |
| Benzocaine 5% | Cobalt chloride 1% |
| Thiomersal 0.1% | Diaminodiphenylmethane 0.5% |
| Carba mix 3% | Mercaptobenzothiazole mix (MBT mix) 2% |
| Quaternium-15 1% | Mercury ammonium chloride 1% |
| Quinoline mix 6% | Imidazolidinyl urea 1% |
| Phenylisopropylparaphenylenediamine 0.1% | p-Toluenediamine 1% |
| Hydrocortisone-17-butyrate 1% (in ethanol) | Budesonide 0.1% |
| Ketoprofen 10% | Latex 0.1% (in water) |
| Tegobetaine 1% (in water) | Euxyl K 400 1.5% |
| Compositae mix 5% | Propylene glycol 10% (in water) |
| Diphenylguanidine 1% | Hexamethylenetetramine 1% |
| Hydroquinone-monobenzylether 1% | Palladium chloride 1% |
| Eugenol 5% | Hydroquinone 1% |
| Mercury 0.5% | Ethylene glycol dimethacrylate 2% |
| Methyl methacrylate 5% | Bisphenol A dimethacrylate 2% |
| Propolis 20% | Sorbic acid 5% |
| Butylated hydroxyanisole 2% | Alpha-tocopherol 20% |
| **(B) Second Patch Test** | |
| Tin 2.5% | Colloidal silver 0.1% |
| Tin chloride 1% | Zinc 2.5% |
| Copper sulfate 1% (in water) | Zinc chloride 2% |
| Silver nitrate 1% (in water) | Zinc sulfate 2% |

inflammation[10,11,12,13,14,15,16,17,18]. Our case appears as a good model of neuroinflammation; the distribution of lesions supports the idea of neuroinflammation involving trigeminal branches that innervate teeth and perioral skin. The spreading of neuroinflammation, through a longer path, to another trigeminal branch not in contact with the triggering agent[2] might explain the new, “delayed” lesions observed during patch test reading. The amalgam seems the most probable trigger, through two possible mechanisms, not mutually exclusive (see top panel of Fig. 3):

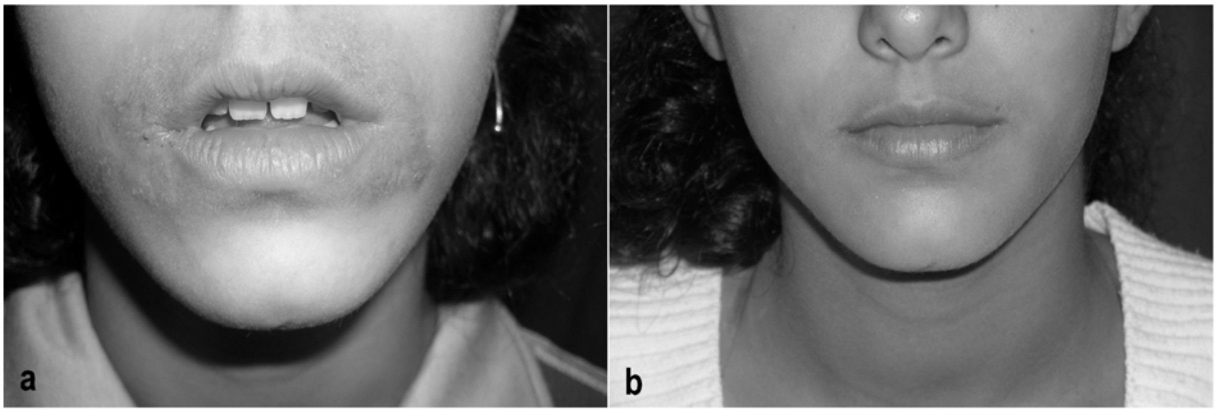

**FIGURE 1.** (a) PD: erythematous-eczematous lesions localized to the lower third of the face, in the perioral area; (b) clinical healing after 4 weeks, persisting at 6 months' follow-up.

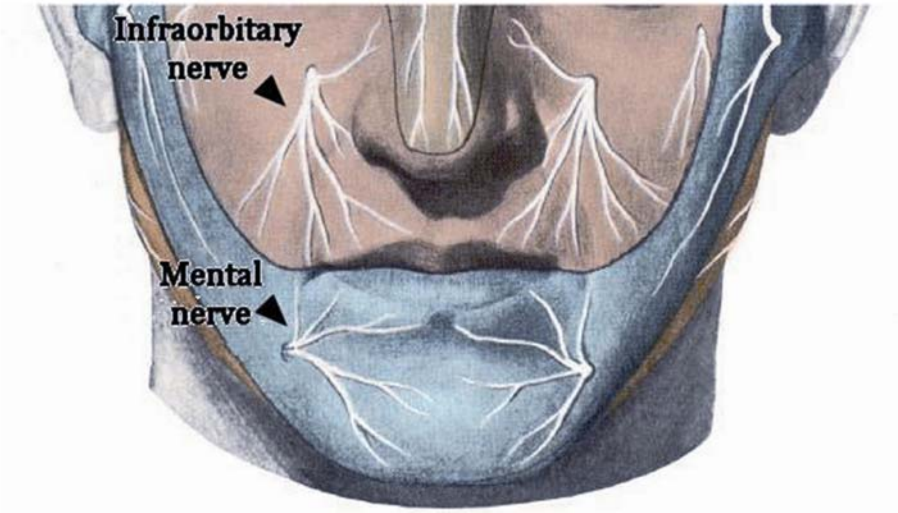

**FIGURE 2**. Distribution of fibers of infraorbitary and mental nerves (sensory branches of trigeminal nerve). (Figure from Balboni GC *et al*., *Anatomia Umana*, vol. 3, 3rd ed, Ed. Ermes, Milan, 1990. Modified with permission).

1. Direct effect of mercury released by the amalgam[19] (and/or methylmercury, synthesized from inorganic mercury by oral bacteria)[20] on neuronal function/excitability and/or cholinergic receptors/pathways — Several papers indicate the possible involvement of the cholinergic system during intoxication by mercury and/or organomercurials[21,22,23]. Although only small quantities of inorganic mercury are released from amalgam, their release in a secluded and restricted microenvironment in close proximity of nerve fibers, such as an amalgam-filled alveolar cavity, could generate local concentrations of mercury/methylmercury sufficiently high enough to affect single nerve branches negatively and trigger neuroinflammation. The neurotoxic effects of organomercurial compounds are partly due to their penetration through cell membranes[23]. Furthermore, methylmercury (highly reactive) can interact with receptors, channels, and enzymes, damaging neuronal membrane functionality and excitability[24]. Indeed, studies show that several types of cholinergic receptors and/or pathways are directly affected by mercury compounds[25]; for example, methylmercury blocks the binding of acetylcholine to the cholinergic nicotinic receptor

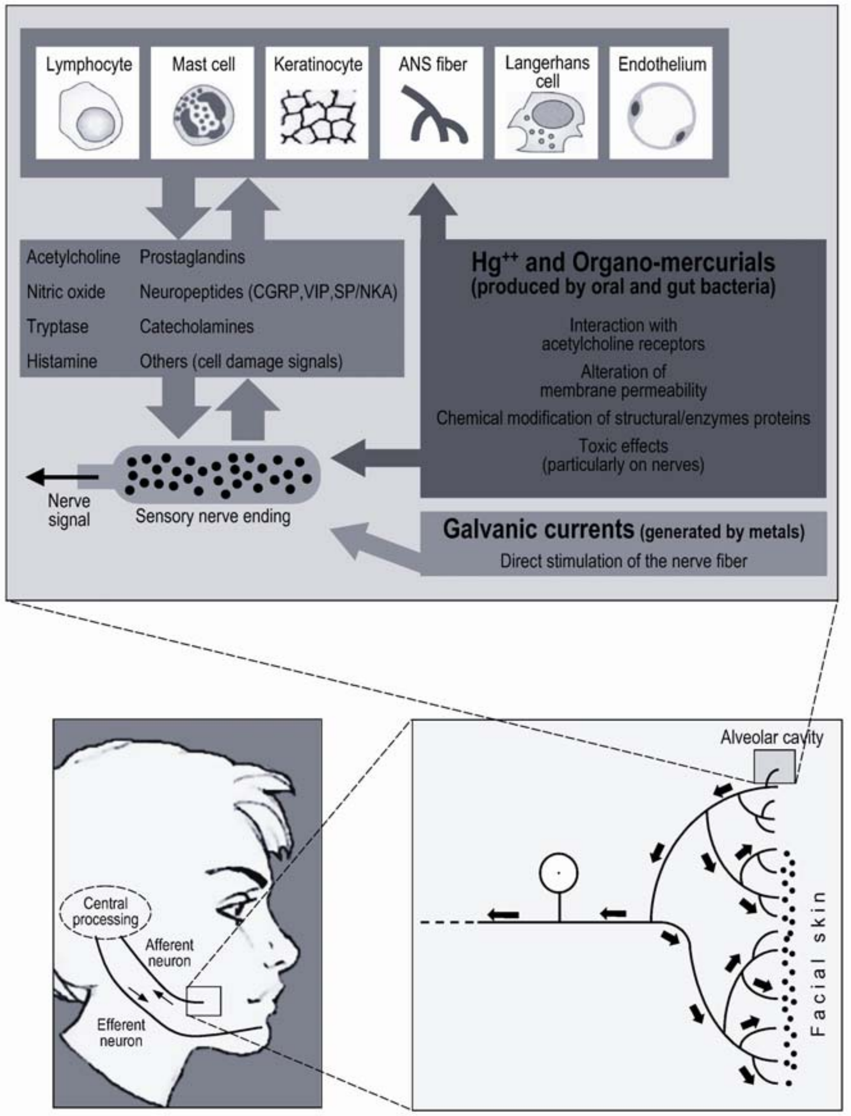

**FIGURE 3.** Schematic representation of possible pathogenic mechanisms involving the cholinergic system in neurogenic inflammation.

of the electric organ of *Torpedo californica*[26]. Also, in *T. californica*, organomercurials can block the activity of the vesicular acetylcholine transporter (VAchT), an enzyme that concentrates acetylcholine in secretory vesicles[27]. Using amino acid sequence comparison, we found highly significant homology between VAchT and its human counterpart, particularly in the transmembrane domain containing the binding point for organomercurials (Fig. 4). On this basis, a negative effect of mercury on the function of human VAchT can be hypothesized and appears worthy of investigation.

2. Allergic reaction to methyl- and ethylmercury — This possibility is supported by a paper by Santucci et al., who demonstrated that the haptenic part of thiomersal is ethylmercury, and that ethyl- and methylmercury are indistinguishable by the immune system from an allergologic perspective[28]. Methylmercury, synthetized from amalgam-derived inorganic mercury by oral and gut bacteria[20], could have elicited type IV hypersensitivity reactions in our patient, leading to release of mediators causing/worsening neuroinflammation.

| | | | |
|---|---|---|---|
| *T. californica* | 369 | IIPLCAL**C**FGIALVDTALLPTLAFLVDI | 396 |
| | | ++ LC L**C**FGIALVDTALLPTLAFLVD+ | |
| *Homo sapiens* | 384 | VVSLCGL**C**FGIALVDTALLPTLAFLVDV | 411 |

**FIGURE 4.** Amino acid sequence homology between the tenth transmembrane segment of the vesicular acetylcholine transporter of *T. californica* and its human counterpart. The symbol "+" indicates a conservative residue substitution. Cysteine-376 of the *T. californica* protein, identified as a binding point for organomercurials (see ref. 27) is marked in bold.

However generated, nerve stimulation could cause perioral skin neuroinflammation through antidromic signal conduction on a specially branching set of nociceptors connecting the alveolar cavity and the face (classic axon reflex)[29,30,31], and/or through an indirect mechanism related to central processing of the trigeminal signal followed by involvement of autonomic parasympathetic fibers[32,33]. These possible paths are represented in the bottom right and bottom left panel of Fig. 3, respectively.

In conclusion, the role of neuroinflammation in PD is worthy of investigation. Apart from the present case, neuroinflammation could play a role, more in general, in the pathogenesis of perioral dermatoses and, possibly, oral lichenoid reactions in patients allergic to dental filling metals. The cholinergic system seems crucial in the communication between oral mucosa, nerves, and the immune system in skin diseases.
